# Editorial: Rhythmic Patterns in Neuroscience and Human Physiology

**DOI:** 10.3389/fnhum.2022.936090

**Published:** 2022-05-25

**Authors:** Nadia Dominici, Marco Iosa, Giuseppe Vannozzi, Daniela De Bartolo

**Affiliations:** ^1^Department of Human Movement Sciences, Amsterdam Movement Sciences, Institute Brain and Behavior Amsterdam, Vrije Universiteit Amsterdam, Amsterdam, Netherlands; ^2^Department of Psychology, Sapienza University of Rome, Rome, Italy; ^3^IRCCS Santa Lucia Foundation, Rome, Italy; ^4^Interuniversity Centre of Bioengineering of the Human Neuromusculoskeletal System, Department of Movement, Human and Health Sciences, University of Rome “Foro Italico”, Rome, Italy

**Keywords:** motor neuroscience, rhythm, coordination dynamics, periodic movements, spatio temporal analysis, harmony

Human movement, as it happens for some other natural phenomena, is characterized by periodicity, with patterns and harmonic structures repeated over time and space. Several mental processes and behaviors have already been described in these terms, that is, through the activation of interconnected neural populations that gives rise to global dynamic states, at the basis of human perception and cognition. Although much research has been directed in this regard, it is still unclear how anatomical connectivity interacts with the neuronal dynamics, from which complex human behavior emerges as rhythmic motor activity.

Accordingly, this Research Topic aimed to collect scientific contributions concerning advances in complex models of brain cells (Rodríguez-Collado and Cristina Rueda) up to motor-behavior control models (Verrelli et al.; Hiraoka; Lindén and Berg; Ulloa), the investigation of coordinated-rhythmic (Benedetto and Baud-Bovy; Bekius et al.; Leach et al.; Pakniyat and Namazi; Dewolf et al.; Den Hartigh et al.; Janzen et al.) and synchronized behaviors (Rosso et al.; Zamm et al.), also including studies conducted on targeted clinical populations of scientific interest (Salesse et al.; Zhao et al.; Rosso et al.).

A total of 16 papers have been accepted including 11 Original Research papers, 2 Mini-Reviews, 1 Review, 1 Case Report, and 1 Hypothesis and Theory.

We have collected scientific contributions that focused on the dynamic systems underlying complex human movements having the property of harmonic spatial and/or temporal structures, such as coordinated and/or synchronized movements.

The ability to produce stable but, at the same time, adaptive behavior raises two constitutive questions. On one hand, it implies the investigation of the mechanisms that are responsible for coordinated actions of the neuromusculoskeletal components of the body that organize themselves temporally and spatially in an ordered and coherent movement pattern. On the other hand, it involves the analysis of cognitive factors such as perceptual processes that guarantee constant feedback on a body in motion and interacting with an environment, in order to select appropriate actions suited to environmental conditions.

In this regard, the paper by Den Hartigh et al. analyzed the concept of resilient motor action providing empirical support between complexity and resilience, and that differential learning supports complexity. Following in the footsteps of Gibson (1979), an alternative approach was that of Leach et al. who investigated what happens to the perception-action dynamics responsible for bimanual coordinated rhythmic movement after differential learning conditions. In this study, although the results differ from predictions, the ecological aspect of the task would seem to guide the transfer. In this regard, in Benedetto and Baud-Bovy a rhythmic tapping task was used to assess the role of perception in the organization of motor representation of the rhythm. These authors found a general effect of rhythm complexity on sensorimotor precision and accuracy speculating that simple rhythms facilitate prediction, requiring less attentional and mnemonical resources.

## Complex Behaviors and Neuronal Dynamics

The review of Ulloa outlined that perception and action are intertwined, at a lower level of organization of the motor system, with feedforward and feedback circuits essential for motor control. This reciprocal interaction is replicated at the highest levels of the organization of the motor system. This entails an assumption of complexity following the hypothesis that the organization observed in human behavior could *ipso facto* imply the existence of a controller, a Central Pattern Generator (CPG), action plan, or internal model that is responsible for its organization and regulation. Usually, the CPG is assumed to be located in the spinal cord and the medulla. Lindén and Berg propose a reinterpretation of this model, focusing on the distribution of firing rates across the spinal neurons population, also suggesting the idea that recurrent inhibition should be considered as embedded in each CPG module (Haghpanah et al., 2017). In the Mini-Review, Hiraoka discussed crossed inhibition during bilateral movement and the anti-phase coordination in rhythmic movements like gait.

Generally speaking, when studying the interlimb coordination dynamics one cannot ignore the investigation of the physiological attractors of movement (Zanone and Kelso, 1997) defined as “attraction of stable movements to maintain robust performance against variability” (Yamamoto et al., 2020). This characteristic is typical of complex dynamic systems such as rhythmic coordinated movements like gait.

In Dewolf et al., neuromuscular control and underlying age-related adjustments were investigated during a level, uphill, and downhill walking as well as stair ascending and descending. Their findings showed subtle age-related differences in all conditions that may potentially reflect systematic age-related adjustments of the neuromuscular control of locomotion across various support surfaces.

In different walking conditions, the foot-off timing undergoes a low variability (Riener et al., 2002), suggesting the possibility that the proportion between stance and swing (60–62% vs. 40–38%) is largely unaffected by external conditions (Lythgo et al., 2011; Iosa et al., 2019). It is interesting that the proportion between the phases of the gait cycle (stride/stance, stance/swing, swing/double support) is stable and coincides with the value of the golden section (Iosa et al., 2013) which has recently been also found in toddlers learning to walk (De Bartolo et al., 2022). In the framework of this temporal point of view, Verrelli et al. proposed a new model of human locomotion, that is based on recursivity, self-similarity, and symmetry of gait assessed through the introduction of a new gait index the *Φ-bonacci gait number*. As hypothesized in Serrao et al. (2017), the golden ratio could allow not only to investigate the harmony of walking but could also represent a physiological attractor of movement.

This concept is not new in the field of neuroscience. (Luria, 1976) wrote about kinetic melodies referring to coordinated and fluid movements, which today are often investigated through the analysis of the coupling of physiological signals to deepen motor control starting from sensorimotor integration (De Bartolo et al., 2020, 2021) (Pakniyat and Namazi; Rosso et al.; Zamm et al.).

Pakniyat and Namazi investigated the variations of the brain and muscle activations while subjects are exposed to different perturbations to walking and standing balance. They found an inverse correlation between changes in brain reactions and muscle activations in standing and walking tasks showing that coordinated behavior such as walking requires greater cerebral but not muscular complexity (Pakniyat and Namazi). These results are in line with the literature on muscle synergies, suggesting that muscle activity may be tailored to task-specific biomechanical needs (Zandvoort et al., 2019).

Recently the literature on the coupling of physiological signals during motor tasks has focused on the phenomenon of auditory-motor (Rosso et al.) and interpersonal synchronization tasks (Zamm et al., 2022). Both of these rely on the physical phenomenon of entrainment firstly reported by Thaut et al. (2015) concerning the frequency locking of two oscillating bodies that can move independently in stable, periodic, or rhythmic cycles (Thaut et al., 2015), but when interacting each other they may assume a common period.

In the study by Zamm et al. (2022) the ability of motor synchronization entrained by music inexperienced musicians performing at spontaneous (uncued) rates was investigated. The authors provided evidence that the dynamics of oscillator coupling are reflected in both behavioral and neural measures of temporal coordination during musical joint action.

Today, this phenomenon of entrainment is the basis of many rehabilitation protocols that use music for motor facilitation, as outlined in the review by Janzen et al. They reported studies using different rehabilitation techniques like rhythmic auditory stimulation, music-supported therapy, therapeutic instrumental music performance, and patterned sensory enhancement and the effectiveness of rhythm and music in restoring motor function in patients with movement-related disorders. In this regard, Rosso et al. proposed a neural outcome measure of auditory-motor coupling that may be useful for rehabilitation purposes.

## Integrated Approaches to Understand Harmonic Movement Patterns

Finally, in this Research Topic, we also selected three papers providing interesting clinical implications.

In a case report, Bekius et al. investigated the development of locomotor patterns and neuromotor control during walking in three very young children with early brain lesions, at risk for developing cerebral palsy (CP). The exploratory longitudinal study reported novel observations by following the children within a period of 1–2 years, presenting different developmental trajectories. The findings revealed differences in maturation of locomotor patterns between children with divergent developmental trajectories.

Zhao et al. reported a study on how restrictedness is manifested in motor behavior. The authors presented an interesting new method by means of entropy analysis to obtain objective behavioral markers of autism spectrum disorder (ASD). This method revealed a lower level of variance in the velocity distribution of participants with ASD, suggesting that children with ASD displayed a more restricted movement compared to typically developing children.

Salesse et al. focused on socio-motor improvisation in individuals with schizophrenia. Using the mirror game paradigm, they recorded hand motions of two people mirroring each other. Results showed that patients exhibited significantly higher difficulties to be synchronized with someone they have to follow, but not when they were leaders of the joint improvisation game.

In light of all these findings, cyclic human movements seem to emerge as a compromise between reliability and adaptable, non-stereotyped patterns. In these terms, human movements should be considered as an orchestrated unique phenomenon, impossible to observe or replicate twice in the same identical way (Stergiou et al., 2006). For this reason, it is essential that the investigation methods are innovative as those proposed in this Research Topic, integrating different techniques in order to characterize the movement in all its complexity.

## Author Contributions

DD: conceptualization. DD and ND: wrote the first draft. GV and MI: revision of the manuscript draft. All authors contributed to manuscript revision, read, and approved the final version.

## Conflict of Interest

The authors declare that the research was conducted in the absence of any commercial or financial relationships that could be construed as a potential conflict of interest.

## Publisher's Note

All claims expressed in this article are solely those of the authors and do not necessarily represent those of their affiliated organizations, or those of the publisher, the editors and the reviewers. Any product that may be evaluated in this article, or claim that may be made by its manufacturer, is not guaranteed or endorsed by the publisher.
